# Color Stability of Composite and Glass Hybrid Restorations in Storage Media Recommended for Avulsed Teeth: An In Vitro Study

**DOI:** 10.3390/ma19143066

**Published:** 2026-07-16

**Authors:** Selvinaz Göksu Apaydın, Akif Demirel, Levent Özer, Merve Berika Kadıoğlu, İlhan Kaya

**Affiliations:** 1 Graduate School of Health Sciences, Ankara University, Altındağ, 06070 Ankara, Turkey; sgapaydin@ankara.edu.tr; 2 Department of Pediatric Dentistry, Faculty of Dentistry, Ankara University, 06560 Ankara, Turkey; akifdemirel@ankara.edu.tr (A.D.); ozer@dentistry.ankara.edu.tr (L.Ö.); 3 Department of Orthodontics, Faculty of Dentistry, Ankara University, 06560 Ankara, Turkey; 4 Department of Oral and Maxillofacial Surgery, Faculty of Dentistry, Bursa Uludağ University, 16120 Bursa, Turkey; ilhankaya@uludag.edu.tr

**Keywords:** tooth avulsion, storage media, color stability, composite resin, glass hybrid restorative, dental trauma

## Abstract

**Highlights:**

- Color change increased over time in all restorative materials and storage media.
- Glass hybrid restorations showed higher ΔE values than composite restorations.
- Storage medium significantly influenced color stability and material performance.
- Storage media may affect both biological and aesthetic outcomes after avulsion.
- The tested composite restorative material demonstrated more predictable short-term color stability.
- Material type should be considered when evaluating avulsed teeth with restorations.

**Abstract:**

Tooth avulsion is one of the most severe dental injuries, and appropriate storage media are essential for preserving avulsed teeth before replantation. However, little is known about the effects of these media on the color stability of existing restorative materials. This in vitro study evaluated the color stability of a composite resin and a glass hybrid restorative material immersed in six storage media commonly recommended for avulsed teeth. A total of 120 disc-shaped specimens were prepared and allocated to dry environment, artificial saliva, tap water, cow’s milk, goat milk, and kefir groups. Color measurements were performed using the CIE L*a*b* system at baseline, 30 min, 1 h, 6 h, 12 h, and 24 h, and color change (ΔE) values were calculated. Data were analyzed using a linear mixed-effects model with restorative material, storage medium, and time as fixed effects and specimen ID as a random effect. Color change increased significantly over time in all groups (*p* < 0.001). Glass hybrid restorations exhibited significantly higher ΔE values than composite restorations across most storage media and time points (*p* < 0.001). Significant material–medium–time interactions were observed (*p* < 0.001), indicating that the effects of storage media varied according to restorative material and exposure duration. Dry storage conditions were generally associated with the highest color changes. Within the limitations of this study, the tested composite restorative material (G-ænial Posterior) demonstrated greater short-term color stability than the tested glass hybrid restorative material (EQUIA Forte HT) in the evaluated storage media.

## 1. Introduction

Dentoalveolar traumatic injuries, particularly those affecting the anterior region, have been demonstrated to result in substantial losses in aesthetics, function and phonation [1,2]. In addition, these injuries can exert significant psychosocial effects on patients. Among the various forms of trauma, avulsion is characterised as the most severe type, necessitating prompt and adequate intervention due to the complete extraction of the tooth from its bony socket [2,3]. During this process, it is of the utmost importance for the avulsed tooth to be stored in a suitable environment until the patient consults a dentist, in order to preserve the periodontal ligament tissues and ensure successful replantation. However, the preservation of both the periodontal vitality and the aesthetic integrity of the avulsed tooth is directly correlated with the treatment’s success, particularly in the anterior region [2,3].

When an avulsed tooth contains an existing restoration, temporary storage before replantation may influence not only periodontal ligament viability but also the optical stability of the restorative material. Consequently, understanding the effect of recommended storage media on restorative material color stability is important for predicting post-traumatic aesthetic outcomes [4,5].

As demonstrated in the extant literature, storage conditions can significantly influence the optical properties and color stability of tooth fragments, with different media inducing varying degrees of change over time, while dehydration itself has been shown to increase enamel opacity and produce clinically significant alterations in tooth shade [6,7,8]. It has been reported that water and dry environments are disadvantageous in this regard; solutions rich in biological content or protein–minerals provide better optical stability [9,10,11]. However, extant studies in the literature focus exclusively on natural dental tissues, and it appears that the question of how and to what extent restorative materials are affected by these storage environments has been largely overlooked. Conversely, restorative materials, due to their composition, demonstrate divergent behavior from dental tissue with regard to water absorption, surface roughness, and light interaction properties. Consequently, the storage environment of an avulsed tooth may result in alterations to the color and translucency of the restoration. This is a significant clinical problem that has a considerable impact on the aesthetic outcomes, particularly in cases where teeth are avulsed in the anterior region and subsequently replanted with restoration. However, the number of studies examining the optical effects of storage solutions on restorative materials remains extremely limited, with a significant gap in the literature [4,5,10,12].

Despite the extensive coverage of the effects of storage media on periodontal structures in the current literature, there is an absence of studies evaluating the effect of these solutions on the optical properties of restorative materials in restored avulsed teeth. Furthermore, there is a paucity of evidence-based data on this subject. Clinically, this situation is of significance, as the color match of the restoration post-replantation may be compromised, necessitating further restorative procedures or resulting in aesthetic dissatisfaction, amongst other potential adverse outcomes. It is imperative to ascertain the optical outcomes of storage media in the context of avulsed tooth restoration, given its significant implications for clinical prediction and treatment planning. In this context, the aim of the present study is to comparatively evaluate the effect of storing composite and glass hybrid restorations in different storage media for varying periods of time on the surface optical properties of these restorative materials. The null hypothesis was that neither restorative material type, storage medium, nor exposure time would significantly influence color change (ΔE) values.

## 2. Materials and Methods

### 2.1. Study Design and Reporting

This in vitro experimental study was conducted in accordance with the CRIS (Checklist for Reporting In-Vitro Studies) guidelines [13]. The study analyzed the color changes (ΔE) of two different restorative materials, resin-based composite (G-ænial Posterior, GC, Tokyo, Japan) and glass hybrid restorative material (EQUIA Forte HT, GC, Tokyo, Japan), in six different avulsed tooth storage media at different time intervals.

### 2.2. Sample Size Calculation

A post hoc sensitivity analysis was performed using G*Power (Version 3.1.9.7, Heinrich Heine University Düsseldorf, Düsseldorf, Germany). Assuming a significance level (α) of 0.05, a target statistical power of 80%, and the present sample size, the study was adequately powered to detect medium-to-large interaction effects. For the primary material × storage medium × time interaction, the minimum detectable effect size was approximately Cohen’s f = 0.42.

### 2.3. Sample Preparation

A total of 120 disc-shaped restorative material samples were prepared for this study. Half of the samples were composite (*n* = 60), and the other half were glass hybrid restorative (*n* = 60) material. All samples were prepared in cast molds made of stainless steel, with a diameter of 10 mm and a thickness of 2 mm, to ensure size standardization.

Preparation of Composite Samples: G-ænial Posterior (GC Corp., Tokyo, Japan) was used for composite samples. The composite material was placed in a single layer in the molds and covered with a Mylar strip to smooth the material surface. A thin glass slide was placed on the strip to remove excess material and level the surface. The samples were polymerized for 20 s using a Woodpecker i-LED Plus LED light device (Guilin Woodpecker Medical Instrument Co., Guilin, China; 1000 mW/cm^2^) in accordance with the manufacturer’s instructions. The probe tip of the device was positioned perpendicular to the sample surface, and a standard glass interface was used to maintain a 1 mm distance between the light source and the material. After polymerization, the upper surfaces of all samples were polished in sequential stages using Sof-Lex Pop-On discs (3M ESPE, Maplewood, MN, USA) to standardize surface roughness.

Preparation of Glass Hybrid Specimens: EQUIA Forte HT (GC Corp., Tokyo, Japan) capsules were used in the preparation of glass hybrid specimens. After the capsules were activated according to the manufacturer’s instructions, they were mixed for 13 s using a Softly8 amalgamator (Micerium S.p.A., Avegno, Italy). The mixed material was placed into the molds using a capsule applicator, ensuring that the entire material was spread homogeneously within the mold. The specimens were left undisturbed during the initial setting time, and after approximately 5 min, the surfaces were coated with EQUIA Forte Coat as recommended by the manufacturer. The coating agent was applied in a thin layer using a microbrush and polymerized for 20 s using an LED light device. This process was performed to increase the surface durability of the material and create a protective layer against moisture.

### 2.4. Storage Media

In the study, a total of six different storage environments were used to evaluate the color stability of restorative materials under different environmental conditions. All specimens were randomly distributed among the six storage media. Each storage medium contained 10 composite and 10 glass hybrid specimens (*n* = 10 per restorative material; total *n* = 20 per storage medium). Samples were allocated to the experimental groups using simple randomization generated by a computer-based random number generator (1:1 allocation). Then, each environment was maintained at 37 °C. To maintain the chemical stability of the solutions and prevent microbial contamination, all storage media were replaced every 30 min (except for the dry environment).

Dry Environment: Samples in this group were stored in an incubator operating at 37 °C under dry conditions without being placed in any liquid solution. This group was used as a control for the natural color change profile of restorative materials in the absence of moisture and liquid contact.

Artificial Saliva: Artificial saliva was prepared to mimic the ionic composition and viscosity of the oral environment and was used in the study to evaluate the stability of the samples in an environment similar to physiological conditions. The formulation was prepared based on the classic composition proposed by McKnight-Hanes & Whitford [14]. Artificial saliva is a solution containing potassium and phosphate buffer systems, calcium and magnesium ions, and sodium carboxymethyl cellulose, mimicking the rheological properties of saliva. All components were dissolved in distilled water, and a homogeneous solution was obtained by balancing the pH.

The artificial saliva solution used in this study was initiated by adding 2.00 g of methyl-p-hydroxybenzoate per 1 L of volume; sodium carboxymethyl cellulose was prepared in an amount of 10.00 g to mimic the viscosity of natural saliva. To mimic the mineral content of saliva, 0.626 g of potassium chloride (KCl), 0.059 g of magnesium chloride hexahydrate (MgCl_2_·6H_2_O), 0.166 g of calcium chloride dihydrate (CaCl_2_·2H_2_O), 0.804 g of potassium hydrogen phosphate (K_2_HPO_4_) 0.804 g, and potassium dihydrogen phosphate (KH_2_PO_4_) 0.326 g were measured and added to the solution. All components were homogenized under a magnetic stirrer until completely dissolved in distilled water; thus, a synthetic saliva was obtained that exhibited properties close to natural saliva in terms of ionic composition and buffering capacity [14].

Tap Water: The water used in this group is standard municipal tap water from the Ankara, Turkey region, and its mineral content, pH level, and ionic components represent natural water sources. Tap water may exhibit different surface interactions with restorative materials due to its mineral components, such as fluoride, calcium, magnesium, and carbonate ions.

Cow’s Milk: Pınar Whole Cow’s Milk (Pınar Süt A.Ş., İzmir, Turkey) was used in this solution.

Goat Milk: Baltalı Goat Milk (Baltalı Gıda, İzmir, Turkey) was used for the goat milk group.

Kefir: Altınkılıç Kefir (Altınkılıç Gıda, İstanbul, Turkey) was used for this group.

### 2.5. Experimental Procedure

After the initial color measurements (T0) were completed, all samples were randomly divided into storage environments. Each sample was placed in containers so that it was completely submerged in the relevant storage liquid, and all containers were stored in an incubator operating at 37 °C. Thus, the intraoral thermal conditions were standardized in the laboratory environment.

To maintain the chemical stability of the environments and prevent microbial contamination during the storage period, all solutions were renewed every 30 min (except for the dry environment). To prevent the accumulation of milk/kefir residues on the surface of the samples, the samples were gently rinsed with pure water before solution changes.

After taking the initial color measurements (T0), all samples were placed in the relevant storage environments, and optical measurements were performed at the following time points:

T1 (30 min): The first follow-up measurement was taken 30 min after the samples were placed in the storage environment.

T2 (1 h): The second measurement was taken at the 1 h mark of the storage process.

T3 (6 h): Samples were kept in the relevant medium for 6 h, and the third measurement was taken.

T4 (12 h): The fourth measurement was performed at the end of the 12th hour.

T5 (24 h): The final measurement was taken on samples stored in the storage environment for 24 h.

A maximum storage period of 24 h was selected because this interval represents a prolonged and clinically challenging scenario that enables evaluation of the cumulative effect of different storage media on the optical stability of restorative materials over time. All measurements were performed by the same researcher, and the samples were placed in a fixed position on the measurement platform to ensure standard device positioning. This process was controlled to distinguish between material surface changes and actual ΔE changes.

To prevent sample loss during inter-time measurements, the same samples were used sequentially for all time points. Each sample was removed from the storage medium at the end of the relevant time interval, gently rinsed with pure water to prevent any milk/kefir or mineral residues that might accumulate on the surface from affecting the measurement results, and lightly cleaned with a cotton pellet. After the optical measurement was completed, the sample was returned to the same storage medium and left until the next time point.

With this protocol, each disk was used in the T1 (30 min), T2 (1 h), T3 (6 h), T4 (12 h), and T5 (24 h) measurements, thus ensuring that the color change of the material over time could be continuously tracked using the same sample. To prevent excessive drying of the surface during cleaning, air spray was applied at low pressure for a short time, and no mechanical abrasive action was performed on the samples. This approach was chosen to reduce variation between samples and to ensure more accurate tracking of the actual color change trend over time.

### 2.6. Optical Measurements

Color measurements of the samples were performed using the VITA Easyshade V (VITA Zahnfabrik, Bad Säckingen, Germany), a spectrophotometer that provides high accuracy in clinical and laboratory applications. Prior to measurement, the device was recalibrated for each measurement point using an automatic calibration block in accordance with the manufacturer’s instructions. To standardize the measurements, all evaluations were performed in an environment with constant lighting conditions, and the device’s probe tip was positioned perpendicular to the sample surface.

Each sample was placed on an opaque and neutral (white) background to minimize reflection. Measurements were taken from the center of the sample, with three consecutive measurements recorded for each sample, and the average of these measurements was used for analysis. To ensure sensor stability between repeated measurements, care was taken to apply constant pressure so that the probe tip was in full contact with the surface.

Color parameters were evaluated in the CIE L*a*b* color space. In this system:

L*: lightness (0 = black, 100 = white)

a*: red-green axis

b*: yellow-blue axis

These values are used to express the color difference (ΔE), which is calculated by comparing the initial measurement (T0) using the following formula:

$$\Delta E_{ab}^{*}=\sqrt{{\left(L_{t}^{*}-L_{0}^{*}\right)}^{2}+{\left(a_{t}^{*}-a_{0}^{*}\right)}^{2}+{\left(b_{t}^{*}-b_{0}^{*}\right)}^{2}}$$

Color differences were calculated using the CIELAB (ΔE*ab) formula because this method remains one of the most widely used approaches for evaluating the color stability of restorative dental materials and facilitates direct comparison with the majority of previously published in vitro studies, including those investigating similar restorative materials.

Before each measurement, samples were rinsed with distilled water, gently cleaned with cotton pellets to prevent milk/kefir residue on the surface, and air-dried briefly to avoid excessive dryness. All measurements were performed by the same operator to minimize operator-related variation. To minimize measurement bias, the operator performing the colour measurements was blinded to the group assignments. Specimens were coded by an independent researcher, and measurements were performed using the coded labels.

**Figure 1:**
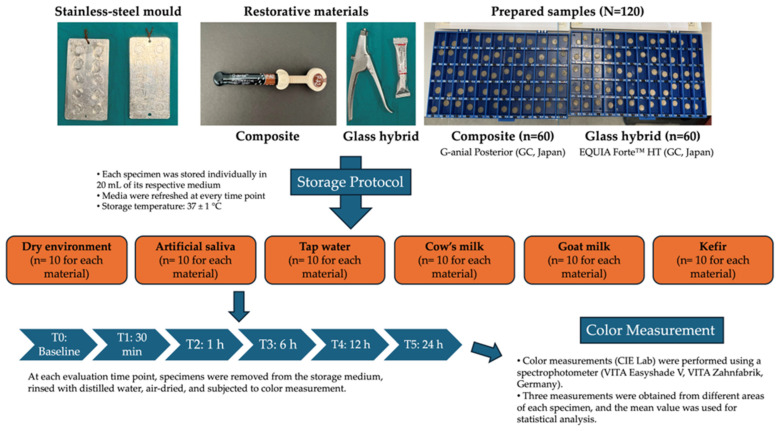
Experimental workflow illustrating specimen preparation, restorative materials, storage protocol, evaluation time points, and color measurement procedures used in the present study.

Color measurements were performed under standardized laboratory conditions at an ambient temperature of approximately 24 °C and a relative humidity of approximately 35%. To minimize the influence of ambient light, measurements were carried out away from direct daylight in a dedicated measurement area surrounded by opaque side panels. The spectrophotometer was calibrated by the laboratory technician according to the manufacturer’s instructions before each measurement session. Between measurement sessions, the specimens remained immersed in their respective storage media in covered containers, thereby minimizing exposure to ambient light. The entire methodological workflow is schematically illustrated in Figure 1.

### 2.7. Statistical Analysis

Statistical analyses were performed using IBM SPSS Statistics for Windows, Version 30.0 (IBM Corp., Armonk, NY, USA). Prior to analysis, the dataset was transformed from wide to long format, with each row representing a single specimen measured at a specific time point. Because repeated measurements were obtained from the same specimens over time, a linear mixed-effects model was applied to account for within-specimen correlation. Time was specified as a categorical fixed factor with five levels (30 min, 1 h, 6 h, 12 h, and 24 h) rather than as a continuous covariate. This approach was chosen because the measurement intervals were unequal and a non-linear pattern of color change over time was considered biologically plausible. Accordingly, the linear mixed-effects model allowed estimation of time-specific effects without assuming a linear relationship between time and color change. An autoregressive [AR(1)] covariance structure was selected for the repeated measures based on the lowest Akaike Information Criterion (AIC). Model assumptions were evaluated using residual diagnostics. The normality of residuals was assessed by visual inspection of normal Q–Q plots together with the Shapiro–Wilk test, whereas homoscedasticity was evaluated by examining plots of standardized residuals against predicted values. The diagnostic procedures did not indicate substantial deviations from model assumptions. Color change (ΔE) was specified as the dependent variable. Restorative material, storage medium, time, and all two-way and three-way interactions among these factors were included as fixed effects, while specimen ID was entered as a random intercept. Model parameters were estimated using maximum likelihood estimation. Type III Wald chi-square tests were used to evaluate the significance of fixed effects. Estimated marginal means (EMMs) and their corresponding 95% confidence intervals were calculated for each material–storage medium–time combination. Pairwise post-hoc comparisons were performed to further investigate significant interaction effects, and *p*-values were adjusted using the Holm–Bonferroni correction to account for multiple testing. Statistical significance was set at *p* < 0.05.

## 3. Results

**Figure 2:**
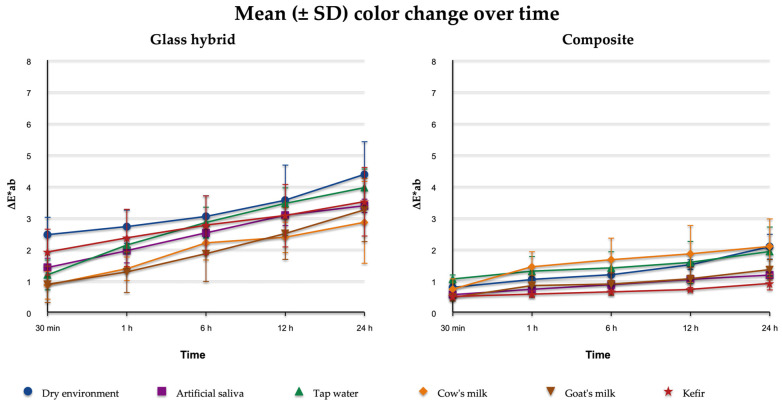
Time-dependent color change (ΔE*ab) of the tested glass hybrid (EQUIA Forte™ HT) and resin composite (G-ænial Posterior) restorative materials immersed in six storage media recommended for avulsed teeth. Data are presented as mean ± standard deviation (SD) at 30 min, 1 h, 6 h, 12 h, and 24 h. Each storage medium is represented by a unique color and marker symbol to improve figure readability. Error bars represent standard deviations. Storage media are identified by both color and marker symbol.

For statistical analyses, storage media were coded numerically to facilitate data management and model construction. Code 1 represented the dry environment, code 2 artificial saliva, code 3 tap water, code 4 cow’s milk, code 5 goat milk, and code 6 kefir. These codes were used throughout the database and statistical analyses while preserving the original storage medium classifications (Table 1). The temporal pattern of color change for each restorative material and storage medium is illustrated in Figure 2. Overall, ΔE values increased progressively over time, with glass hybrid restorations demonstrating consistently higher color change than composite restorations across most storage conditions (Figure 2).

The linear mixed-effects model revealed significant effects of restorative material, storage medium, and time on color change values. In addition, significant interaction effects were observed between the investigated variables, indicating that the influence of storage media on color stability varied according to both restorative material type and exposure time. These findings demonstrate that color change is a multifactorial phenomenon influenced by the combined effects of material composition, storage environment, and duration of exposure (Table 2).

Descriptive analysis demonstrated a progressive increase in color change (ΔE) values over time in both restorative materials and across all storage media. Glass hybrid specimens consistently exhibited higher mean ΔE values than composite specimens throughout the observation period. For glass hybrid restorations, the greatest color changes were generally observed in the dry environment, tap water, and kefir groups, whereas cow’s milk and goat milk showed comparatively lower ΔE values. Composite specimens displayed substantially lower color changes in all storage media, with the lowest values generally observed in the kefir, artificial saliva, and goat milk groups. At the final observation point (24 h), the highest mean ΔE value was recorded for glass hybrid specimens stored in a dry environment (4.37 ± 1.04), while the lowest value was observed for composite specimens stored in kefir (0.91 ± 0.20). Overall, the descriptive findings indicate that both storage medium and exposure duration may influence the color stability of restorative materials, with glass hybrid restorations appearing more susceptible to color alteration than composite restorations (Table 3).

The linear mixed-effects analysis showed that restorative material, storage medium, and time each had a significant effect on color change (ΔE) values (*p* < 0.001 for all effects). In addition to these main effects, significant interaction effects were also identified between the investigated variables. Both the material × storage medium interaction and the material × time interaction were statistically significant (*p* < 0.001), indicating that the magnitude of color change differed according to the restorative material used and that this difference varied over time and across storage media. Furthermore, a significant storage medium × time interaction was observed (*p* < 0.001), suggesting that the influence of the storage environment on color stability was not constant throughout the observation period. Most importantly, the three-way interaction between restorative material, storage medium, and time was also significant (Wald χ^2^ = 56.764, *p* < 0.001). This finding indicates that the temporal pattern of color change depended simultaneously on both the type of restorative material and the storage medium in which the specimens were kept. Therefore, the effects of storage media on color stability should be interpreted in relation to both material type and exposure duration rather than as isolated factors (Table 4).

Because a significant three-way interaction was identified in the mixed-effects model, estimated marginal means were examined to better understand the pattern of color change across restorative materials, storage media, and time intervals. Overall, ΔE values increased progressively over time in all groups. Glass hybrid restorations consistently exhibited higher estimated color change values than composite restorations regardless of storage medium. Among glass hybrid specimens, the highest estimated ΔE values were observed in the dry environment throughout the study period, increasing from 2.46 at 30 min to 4.37 at 24 h. Relatively high values were also observed in the tap water and kefir groups. In contrast, cow’s milk and goat milk generally showed lower ΔE values, particularly during the earlier observation periods. For composite restorations, color changes remained lower across all storage media. The highest estimated ΔE values at 24 h were observed in the dry environment and cow’s milk groups (both ΔE = 2.08), whereas kefir consistently demonstrated the lowest values throughout the study period, increasing only from 0.50 at 30 min to 0.91 at 24 h. Taken together, these findings indicate that color stability was influenced not only by the restorative material itself but also by the interaction between storage medium and exposure time (Table 5).

Post-hoc analyses were performed to compare composite and glass hybrid restorations within each storage medium at each time point. In most storage media, composite restorations demonstrated significantly lower ΔE values than glass hybrid restorations, as reflected by the negative mean differences observed throughout the study period. In the dry environment, artificial saliva, and kefir groups, significant differences between the two restorative materials were present at all time points after Holm–Bonferroni adjustment (adjusted *p* < 0.001 for all comparisons). A similar pattern was observed in the goat milk group from 6 h onwards, where glass hybrid restorations showed progressively greater color change than composite restorations. In the tap water group, no significant difference was observed at 30 min; however, significant differences emerged from 1 h onwards and became more pronounced over time. Likewise, in the cow’s milk group, the two materials exhibited comparable color stability during the early observation periods, whereas significant differences were detected at 6 h and 24 h. Overall, these findings indicate that composite restorations were generally more resistant to color alteration than glass hybrid restorations, although the magnitude and timing of these differences varied according to the storage medium (Table 6).

To further explore the significant interaction effects identified in the mixed-effects model, pairwise comparisons between storage media were performed separately for each restorative material and time interval. Only comparisons that remained significant after Holm–Bonferroni adjustment are presented. For glass hybrid restorations, significant differences between storage media were already evident at the earliest observation period (30 min). In particular, specimens stored in a dry environment exhibited significantly greater color change than those stored in artificial saliva, cow’s milk, goat milk, and kefir. Similar patterns persisted throughout the study period, with dry environment and tap water generally producing higher ΔE values than milk-based media. Comparisons involving cow’s milk and goat milk frequently demonstrated lower color changes, supporting their relatively protective effect on color stability. For composite restorations, fewer significant differences were observed during the early observation periods. However, as exposure time increased, storage-medium-dependent differences became more apparent. At later time points, kefir generally exhibited lower ΔE values than several other storage media, whereas dry environment, tap water, and cow’s milk were more frequently associated with higher color change values. Overall, the post-hoc analyses confirmed that the effect of storage medium on color stability differed according to restorative material type and became more pronounced with increasing storage duration (Table 7).

## 4. Discussion

The present study evaluated the effects of different storage media on the color stability of composite and glass hybrid restorative materials over time using a repeated-measures mixed-effects approach. The results demonstrated that color change was significantly influenced by restorative material, storage medium, and exposure time. Furthermore, significant interaction effects indicated that the impact of a storage medium on color stability varied according to both the restorative material and the duration of exposure. Overall, glass hybrid restorations exhibited greater color changes than composite restorations, while color change increased progressively with increasing storage time. Accordingly, the null hypothesis was rejected, as restorative material type, storage medium, exposure time, and their interactions significantly influenced ΔE values.

G-ænial Posterior was selected as the representative resin composite material, whereas EQUIA Forte HT was chosen as the glass hybrid restorative material. Previous studies have reported satisfactory color stability and clinically acceptable ΔE values for different members of the G-ænial family following exposure to staining solutions and artificial ageing protocols [15,16]. In the present study, composite specimens consistently exhibited lower color change values than glass hybrid specimens across most storage media and time intervals. This finding may be related to the lower water sorption and greater color stability generally associated with resin-based composite materials. However, because physicochemical mechanisms such as water sorption, ion diffusion, or protein adsorption were not directly evaluated, these explanations should be regarded as plausible interpretations rather than experimentally confirmed mechanisms. In contrast, EQUIA Forte HT contains reactive glass particles and an acid–base setting matrix that may be more susceptible to water uptake and surface alterations during prolonged exposure to aqueous environments [17,18,19]. Although composite restorations are more commonly encountered in anterior teeth affected by avulsion injuries, glass hybrid materials are increasingly used in pediatric dentistry for cervical lesions and selected posterior restorations. Therefore, comparing these two restorative materials under conditions simulating common avulsion storage media provides clinically relevant information regarding their aesthetic performance following accidental tooth trauma. In addition to optical stability, the overall clinical performance of restorative materials is influenced by their mechanical behavior, structural characteristics, and physicochemical properties. Previous studies have shown that material composition and microstructural characteristics influence not only mechanical performance but also the optical and aging behavior of restorative materials under different clinical conditions [20]. Likewise, previous investigations comparing restorative materials have demonstrated that different material classes exhibit distinct mechanical performance, emphasizing the importance of comprehensive material evaluation rather than relying on a single property [21]. Therefore, the differences in color stability observed in the present study should be interpreted as one aspect of the overall material performance rather than as an isolated property.

The present study demonstrated that color change was significantly influenced by restorative material, storage medium, and exposure time. In addition, the significant interaction effects observed in the mixed-effects model indicated that the impact of a given storage medium varied according to both the restorative material and the duration of exposure. Overall, ΔE values increased progressively over time in all groups, confirming that color stability is a time-dependent phenomenon. Glass hybrid restorations consistently exhibited greater color change than composite restorations across most storage media and observation periods. Among the glass hybrid specimens, the highest ΔE values were generally observed in the dry environment and tap water groups, whereas cow’s milk and goat milk were associated with comparatively lower color changes. In contrast, composite restorations showed lower and more stable ΔE values throughout the study period, with particularly low color changes observed in the kefir and goat milk groups. From a clinical perspective, most color changes remained within or close to the range of commonly reported acceptability thresholds during the early observation periods. However, several glass hybrid groups approached or exceeded these thresholds after prolonged exposure, particularly under dry conditions. These findings suggest that the aesthetic consequences of storage media may become clinically relevant depending on both the restorative material present and the duration of storage [22,23].

From a clinical perspective, interpretation of color differences should consider established acceptability thresholds reported in the literature. Alghazali et al. suggested an acceptability threshold of approximately 4.2 ΔE units, whereas Khashayar et al. reported that a ΔE value of approximately 3.7 is among the most frequently used clinical acceptability thresholds in dentistry [22,23]. In the present study, the glass hybrid material stored in a dry environment reached a mean ΔE value of 4.37 ± 1.04 after 24 h, exceeding the commonly accepted threshold values. Similarly, glass hybrid specimens stored in tap water (3.95 ± 0.60) approached or exceeded commonly reported acceptability thresholds, whereas kefir (3.50 ± 1.09) remained close to the lower threshold values. In contrast, all composite groups remained below these thresholds throughout the experimental period. These findings suggest that storage conditions may have clinically relevant aesthetic implications, particularly for glass hybrid restorations exposed to prolonged storage periods.

Although few studies have investigated the optical behaviour of restorative materials in storage media recommended for avulsed teeth, the findings of the present study are broadly consistent with previous research on color stability and water-related degradation of restorative materials. The mixed-effects analysis demonstrated that restorative material, storage medium, and exposure time all significantly influenced color change, with glass hybrid restorations generally exhibiting higher ΔE values than composite restorations. Several studies have reported that conventional and resin-modified glass ionomer-based materials exhibit greater water sorption and solubility than resin composites, which may adversely affect their optical stability [8]. It has been proposed that increased water sorption may promote surface degradation and facilitate pigment diffusion into restorative materials, thereby contributing to color alteration. Similarly, Huang et al. [12] reported a positive relationship between water sorption, solubility, and color change in resin-based restorative materials. Because the present study did not directly evaluate physicochemical mechanisms such as water sorption, ion diffusion, protein adsorption, or dehydration, these explanations should be interpreted as plausible mechanisms based on previous literature rather than experimentally confirmed findings. Nevertheless, these mechanisms may partly explain the greater color alterations observed in the glass hybrid specimens.

The current findings are also in agreement with previous investigations comparing resin-based and glass ionomer-based restorative materials. Hotwani et al. [24] demonstrated that resin-based giomer materials exhibited significantly lower color change than resin-modified glass ionomer materials when exposed to commonly consumed children’s beverages. Likewise, higher discoloration values have been reported for glass hybrid materials such as EQUIA Forte following exposure to acidic solutions and staining media [25]. Although surface coating agents may improve the short-term performance of glass ionomer-based materials by reducing surface porosity, their protective effect appears to be influenced by the type of material, storage environment, and duration of exposure. In this regard, Çarıkçıoğlu [26] reported no significant long-term color advantage of coated glass ionomer materials compared with uncoated specimens after ageing procedures.

An additional finding of the present study was that the effect of storage medium was not uniform across restorative materials and changed over time, as evidenced by the significant interaction effects observed in the mixed-effects model. This observation suggests that color stability is determined not only by intrinsic material properties but also by the dynamic interaction between material composition, environmental conditions, and exposure duration. Although the present study evaluated restorative materials rather than natural dental tissues, the findings are conceptually consistent with previous studies emphasizing the importance of storage conditions for maintaining optical integrity. Tuzuner et al. [6] reported that tooth fragments stored in dry conditions exhibited marked color changes due to dehydration, whereas storage in milk or saliva reduced these alterations. While that study focused on natural enamel and dentin rather than restorative materials, both investigations highlight the critical influence of storage media on short-term aesthetic outcomes.

The present study provides novel information regarding the short-term optical behaviour of restorative materials under conditions relevant to avulsed teeth. Unlike most color stability studies, which primarily evaluate the effects of commonly consumed staining agents such as coffee, tea, cola, red wine, pediatric syrups, or energy drinks on restorative materials [27], the current investigation focused on storage media that are routinely recommended following tooth avulsion. This distinction is clinically relevant because the primary purpose of these media is to preserve periodontal ligament viability rather than to act as staining challenges. Although storage media such as milk and saliva have occasionally been used in laboratory studies, their effects on restorative material color stability have received limited attention. Furthermore, systematic reviews evaluating storage media for avulsed teeth have traditionally focused on periodontal ligament cell survival and healing outcomes, with little consideration given to the aesthetic performance of existing restorations [28]. Consequently, there remains a substantial gap in the literature regarding how storage environments may simultaneously influence both biological and restorative outcomes following avulsion injuries. From a clinical perspective, this issue is particularly important because many avulsed teeth, especially in children and adolescents, may already contain restorative materials at the time of trauma. Therefore, understanding the influence of recommended storage media on the optical stability of restorative materials may contribute to more comprehensive post-traumatic treatment planning and aesthetic outcome prediction. The findings of the present study help address this underexplored aspect of dental traumatology and may provide a foundation for future investigations evaluating both periodontal and restorative outcomes within the same experimental model.

The findings of the present study suggest that the aesthetic performance of restorative materials following tooth avulsion may be influenced not only by the restorative material itself but also by the storage medium used and the duration of storage. Although many avulsed teeth are replanted within a considerably shorter period, delayed replantation is still encountered in clinical practice because of late presentation, transportation difficulties, or lack of immediate access to dental care. Therefore, evaluating a prolonged storage period provides clinically relevant information regarding the cumulative effects of different storage media under unfavorable conditions. The significant interaction effects observed in the mixed-effects model indicate that no single storage medium produced identical effects across all restorative materials. Nevertheless, dry storage conditions were generally associated with greater color change, particularly in glass hybrid restorations, whereas milk-based media and artificial saliva tended to be associated with lower ΔE values under several experimental conditions. These findings suggest that the type of restoration present on an avulsed tooth may be considered when evaluating the potential aesthetic consequences of temporary storage. However, aesthetic considerations should always remain secondary to the preservation of periodontal ligament cell viability and overall treatment prognosis. Importantly, Hank’s Balanced Salt Solution (HBSS), which is widely regarded as the reference storage medium for avulsed teeth, was not included in the present study. Therefore, the current findings should not be interpreted as recommendations regarding the biological suitability of storage media, but rather as an evaluation of their potential influence on the short-term color stability of restorative materials.

The present study has several strengths. First, both composite and glass hybrid restorative materials were evaluated under identical experimental conditions using six storage media commonly recommended for avulsed teeth. Second, specimen preparation and surface finishing procedures were standardized using stainless steel molds and a single operator, thereby reducing methodological variability. Third, color measurements were performed using a spectrophotometer and the CIE Lab* color system, with three consecutive measurements obtained for each specimen to enhance measurement reliability. Finally, multiple clinically relevant time points within the first 24 h after trauma were evaluated, allowing the temporal pattern of color change to be assessed in detail.

Nevertheless, several limitations should be considered when interpreting the findings. First, the flat, polished disc specimens used in this in vitro study do not fully reproduce the anatomical morphology, restoration geometry, finishing and polishing procedures, marginal configuration, or the complex optical behaviour of restored teeth under clinical conditions. Furthermore, the influence of enamel and dentin substrates, adhesive interfaces, and irregular fracture surfaces cannot be adequately simulated using standardized disc specimens. Factors such as enamel and dentin structure, adhesive interfaces, restoration geometry, and fracture-line characteristics could not be reproduced within the experimental model. Second, only short-term color changes during the first 24 h were evaluated; therefore, the long-term effects of storage media remain unknown. Thermal cycling, mechanical loading, and artificial ageing procedures were not included and may influence color stability under clinical conditions. Third, only two restorative materials and a single shade were investigated. Different restorative systems, shades, translucencies, and surface treatments may exhibit different responses to the storage media evaluated in this study. Although the selected materials are widely used in clinical practice, restorative materials from different manufacturers may differ considerably in resin matrix composition, filler characteristics, water sorption behavior, and optical properties. Therefore, the present findings should not be generalized to all resin composites or all glass hybrid restorative materials, and further studies evaluating a broader range of materials are warranted. Finally, although significant differences were detected under the present experimental conditions, future studies with larger sample sizes are warranted to further strengthen the external validity and generalizability of these findings. Another limitation of the present study is that surface characteristics of the restorative materials, such as surface roughness or micromorphological alterations evaluated by scanning electron microscopy (SEM), were not assessed. Because changes in surface integrity may contribute to discoloration, simultaneous evaluation of optical and surface properties would provide a more comprehensive understanding of the mechanisms underlying color change. Another limitation of the present study is that the pH values of the storage media were not measured during the experimental period. Although the storage media were used according to standardized protocols, variations in pH may influence the optical behavior and degradation of restorative materials. Therefore, the potential contribution of pH to the observed color changes could not be evaluated directly in the present study. In addition, although a post hoc sensitivity analysis indicated that the study had adequate statistical power, an a priori sample size calculation would have provided a stronger methodological basis for determining the required sample size. This should be considered in future investigations.

Future studies should expand upon the findings of the present investigation by evaluating a broader range of restorative materials, including contemporary nano-hybrid, bulk-fill, and bioactive restorative systems commonly used in pediatric and restorative dentistry. In addition, the inclusion of storage media such as Hank’s Balanced Salt Solution (HBSS), physiological saline, and other commercially available dairy products would provide a more comprehensive understanding of the relationship between storage conditions and restorative material performance. Because color stability is influenced by multiple physical and chemical factors, future research should also investigate parameters such as surface roughness, microhardness, water sorption, solubility, mass loss, and ion release alongside color change measurements. Furthermore, the incorporation of thermal cycling, mechanical loading, and long-term ageing protocols would allow experimental conditions to more closely resemble the clinical environment. Also, future studies should combine color measurements with surface characterization techniques, such as surface roughness analysis and SEM, to better elucidate the mechanisms responsible for discoloration. In addition, further studies should also evaluate the influence of physicochemical properties of storage media, including pH, on the color stability and surface characteristics of restorative materials. Finally, future experimental models should aim to evaluate both periodontal and restorative outcomes simultaneously. Studies combining periodontal ligament cell viability assessments with the optical behaviour of restorative materials in ex-vivo tooth models may provide a more comprehensive understanding of post-traumatic storage strategies and their potential implications for both biological and aesthetic treatment outcomes.

## 5. Conclusions

This in vitro study demonstrated that both restorative material type and storage medium influenced the short-term color stability of the tested restorative materials following immersion in storage media recommended for avulsed teeth. Color change increased progressively over time in both materials; however, under the present experimental conditions, the tested resin composite (G-ænial Posterior) exhibited lower ΔE values than the tested glass hybrid restorative material (EQUIA Forte HT) in most storage media. The findings also suggest that storage media may affect not only periodontal ligament preservation but also the optical behavior of existing restorations on avulsed teeth. Nevertheless, because only one resin composite and one glass hybrid restorative material were evaluated, the results should be interpreted as material-specific and should not be generalized to all restorative materials within these categories. Overall, this study provides novel evidence regarding the interaction between storage media and restorative material color stability in simulated avulsion conditions and may serve as a foundation for future investigations incorporating a broader range of restorative materials, longer aging protocols, and additional surface and physicochemical analyses.

## Figures and Tables

**Table 1:** Coding system used for storage media included in the study.

Code	Storage Medium
1	Dry environment
2	Artificial saliva
3	Tap water
4	Cow’s milk
5	Goat milk
6	Kefir

**Table 2:** Model characteristics and variance estimate of the linear mixed-effects model used in the study.

Parameter	Value
Observations	600
Specimens	120
Repeated measurements per specimen	5
Model likelihood method	Maximum likelihood (ML)
Random effect	Specimen ID
Residual variance (scale)	0.0932
Random intercept variance	0.2065
Convergence	Yes

**Table 3:** Descriptive ΔE values (mean ± standard deviation) according to restorative material, storage medium, and time interval.

Material	Medium	30 min	1 h	6 h	12 h	24 h
Glass hybrid	Dry environment	2.46 ± 0.55	2.71 ± 0.52	3.04 ± 0.66	3.55 ± 1.12	4.37 ± 1.04
Glass hybrid	Artificial saliva	1.42 ± 0.29	1.95 ± 0.39	2.52 ± 0.28	3.08 ± 0.33	3.37 ± 0.21
Glass hybrid	Tap water	1.18 ± 0.47	2.13 ± 0.25	2.84 ± 0.49	3.45 ± 0.49	3.95 ± 0.60
Glass hybrid	Cow’s milk	0.85 ± 0.44	1.38 ± 0.37	2.20 ± 0.54	2.37 ± 0.48	2.85 ± 1.30
Glass hybrid	Goat milk	0.88 ± 0.58	1.27 ± 0.65	1.85 ± 0.88	2.49 ± 0.82	3.24 ± 1.01
Glass hybrid	Kefir	1.91 ± 0.73	2.36 ± 0.91	2.76 ± 0.93	3.06 ± 0.99	3.50 ± 1.09
Composite	Dry environment	0.79 ± 0.20	1.03 ± 0.21	1.18 ± 0.18	1.50 ± 0.16	2.08 ± 0.39
Composite	Artificial saliva	0.55 ± 0.11	0.72 ± 0.13	0.87 ± 0.14	1.04 ± 0.14	1.18 ± 0.07
Composite	Tap water	1.05 ± 0.13	1.29 ± 0.47	1.40 ± 0.52	1.58 ± 0.66	1.92 ± 0.78
Composite	Cow’s milk	0.73 ± 0.33	1.44 ± 0.48	1.66 ± 0.69	1.85 ± 0.90	2.08 ± 0.88
Composite	Goat milk	0.46 ± 0.11	0.84 ± 0.33	0.89 ± 0.35	1.06 ± 0.37	1.34 ± 0.33
Composite	Kefir	0.50 ± 0.10	0.57 ± 0.10	0.64 ± 0.06	0.72 ± 0.07	0.91 ± 0.20

**Table 4:** Type III Wald tests of fixed effects obtained from the linear mixed-effects model evaluating the effects of restorative material, storage medium, time, and their interactions on color change (ΔE).

Effect	Wald Chi-Square	df	*p*
Material	250.356	1	<0.001
Storage medium	41.401	5	<0.001
Time	1747.258	4	<0.001
Material × Storage medium	42.572	5	<0.001
Material × Time	315.345	4	<0.001
Storage medium × Time	93.655	20	<0.001
Material × Storage medium × Time	56.764	20	<0.001

**Table 5:** Estimated marginal mean (EMM) ΔE values with 95% confidence intervals according to restorative material, storage medium, and time interval.

Material	Medium	30 min	1 h	6 h	12 h	24 h
Glass hybrid	Dry environment	2.46 (2.12, 2.80)	2.71 (2.37, 3.05)	3.04 (2.70, 3.38)	3.55 (3.21, 3.89)	4.37 (4.03, 4.71)
Glass hybrid	Artificial saliva	1.42 (1.08, 1.75)	1.95 (1.61, 2.29)	2.52 (2.18, 2.85)	3.08 (2.74, 3.41)	3.37 (3.03, 3.71)
Glass hybrid	Tap water	1.18 (0.84, 1.52)	2.13 (1.79, 2.47)	2.84 (2.50, 3.18)	3.45 (3.11, 3.79)	3.95 (3.61, 4.29)
Glass hybrid	Cow’s milk	0.85 (0.51, 1.19)	1.38 (1.04, 1.72)	2.20 (1.86, 2.54)	2.37 (2.03, 2.71)	2.85 (2.51, 3.19)
Glass hybrid	Goat milk	0.88 (0.54, 1.22)	1.27 (0.93, 1.61)	1.85 (1.51, 2.19)	2.49 (2.15, 2.83)	3.24 (2.90, 3.58)
Glass hybrid	Kefir	1.91 (1.57, 2.25)	2.36 (2.02, 2.69)	2.76 (2.42, 3.10)	3.06 (2.72, 3.40)	3.50 (3.16, 3.84)
Composite	Dry environment	0.79 (0.45, 1.12)	1.03 (0.69, 1.37)	1.18 (0.84, 1.52)	1.50 (1.16, 1.84)	2.08 (1.74, 2.42)
Composite	Artificial saliva	0.55 (0.21, 0.89)	0.72 (0.38, 1.06)	0.87 (0.53, 1.21)	1.04 (0.70, 1.38)	1.18 (0.84, 1.51)
Composite	Tap water	1.05 (0.71, 1.39)	1.29 (0.96, 1.63)	1.40 (1.06, 1.74)	1.58 (1.24, 1.92)	1.92 (1.58, 2.26)
Composite	Cow’s milk	0.73 (0.39, 1.06)	1.44 (1.10, 1.78)	1.66 (1.32, 2.00)	1.85 (1.51, 2.19)	2.08 (1.74, 2.42)
Composite	Goat milk	0.46 (0.12, 0.80)	0.84 (0.50, 1.18)	0.89 (0.55, 1.23)	1.06 (0.72, 1.40)	1.34 (1.00, 1.68)
Composite	Kefir	0.50 (0.16, 0.83)	0.57 (0.23, 0.91)	0.64 (0.30, 0.98)	0.72 (0.38, 1.06)	0.91 (0.57, 1.24)

**Table 6:** Holm–Bonferroni adjusted post-hoc comparisons between composite and glass hybrid restorative materials within each storage medium and time interval.

Medium	Time	Mean Difference (Composite-Glass Hybrid)	SE	z	*p*	Adjusted *p* (Holm)
Dry environment	30 min	−1.671	0.245	−6.827	<0.001	<0.001
Dry environment	1 h	−1.680	0.245	−6.862	<0.001	<0.001
Dry environment	6 h	−1.853	0.245	−7.569	<0.001	<0.001
Dry environment	12 h	−2.050	0.245	−8.373	<0.001	<0.001
Dry environment	24 h	−2.291	0.245	−9.356	<0.001	<0.001
Artificial saliva	30 min	−0.866	0.245	−3.538	<0.001	0.004
Artificial saliva	1 h	−1.230	0.245	−5.024	<0.001	<0.001
Artificial saliva	6 h	−1.645	0.245	−6.721	<0.001	<0.001
Artificial saliva	12 h	−2.035	0.245	−8.313	<0.001	<0.001
Artificial saliva	24 h	−2.197	0.245	−8.973	<0.001	<0.001
Tap water	30 min	−0.133	0.245	−0.543	0.587	1.000
Tap water	1 h	−0.831	0.245	−3.395	<0.001	0.006
Tap water	6 h	−1.444	0.245	−5.898	<0.001	<0.001
Tap water	12 h	−1.871	0.245	−7.641	<0.001	<0.001
Tap water	24 h	−2.029	0.245	−8.287	<0.001	<0.001
Cow’s milk	30 min	−0.127	0.245	−0.519	0.604	1.000
Cow’s milk	1 h	0.060	0.245	0.246	0.806	1.000
Cow’s milk	6 h	−0.534	0.245	−2.182	0.029	0.204
Cow’s milk	12 h	−0.524	0.245	−2.141	0.032	0.204
Cow’s milk	24 h	−0.765	0.245	−3.124	0.002	0.014
Goat milk	30 min	−0.417	0.245	−1.702	0.089	0.393
Goat milk	1 h	−0.431	0.245	−1.759	0.079	0.393
Goat milk	6 h	−0.964	0.245	−3.937	<0.001	<0.001
Goat milk	12 h	−1.436	0.245	−5.867	<0.001	<0.001
Goat milk	24 h	−1.901	0.245	−7.764	<0.001	<0.001
Kefir	30 min	−1.412	0.245	−5.768	<0.001	<0.001
Kefir	1 h	−1.787	0.245	−7.300	<0.001	<0.001
Kefir	6 h	−2.116	0.245	−8.642	<0.001	<0.001
Kefir	12 h	−2.345	0.245	−9.577	<0.001	<0.001
Kefir	24 h	−2.598	0.245	−10.611	<0.001	<0.001

**Table 7:** Holm–Bonferroni adjusted pairwise comparisons between storage media within each restorative material and time interval.

Material	Time	Comparison	Mean Difference	SE	z	*p*	Adjusted *p* (Holm)
Glass hybrid	30 min	Dry environment-Artificial saliva	1.041	0.245	4.252	<0.001	<0.001
Glass hybrid	30 min	Dry environment-Tap water	1.277	0.245	5.216	<0.001	<0.001
Glass hybrid	30 min	Dry environment-Cow’s milk	1.604	0.245	6.551	<0.001	<0.001
Glass hybrid	30 min	Dry environment-Goat milk	1.578	0.245	6.445	<0.001	<0.001
Glass hybrid	30 min	Tap water-Kefir	−0.728	0.245	−2.973	0.003	0.027
Glass hybrid	30 min	Cow’s milk-Kefir	−1.055	0.245	−4.308	<0.001	<0.001
Glass hybrid	30 min	Goat milk-Kefir	−1.029	0.245	−4.202	<0.001	<0.001
Glass hybrid	1 h	Dry environment-Artificial saliva	0.764	0.245	3.119	0.002	0.018
Glass hybrid	1 h	Dry environment-Cow’s milk	1.336	0.245	5.459	<0.001	<0.001
Glass hybrid	1 h	Dry environment-Goat milk	1.444	0.245	5.897	<0.001	<0.001
Glass hybrid	1 h	Artificial saliva-Goat milk	0.680	0.245	2.777	0.005	0.044
Glass hybrid	1 h	Tap water-Cow’s milk	0.749	0.245	3.061	0.002	0.020
Glass hybrid	1 h	Tap water-Goat milk	0.856	0.245	3.499	<0.001	0.005
Glass hybrid	1 h	Cow’s milk-Kefir	−0.979	0.245	−3.997	<0.001	<0.001
Glass hybrid	1 h	Goat milk-Kefir	−1.086	0.245	−4.435	<0.001	<0.001
Glass hybrid	6 h	Dry environment-Cow’s milk	0.839	0.245	3.429	<0.001	0.007
Glass hybrid	6 h	Dry environment-Goat milk	1.184	0.245	4.838	<0.001	<0.001
Glass hybrid	6 h	Tap water-Goat milk	0.991	0.245	4.049	<0.001	<0.001
Glass hybrid	6 h	Goat milk-Kefir	−0.905	0.245	−3.698	<0.001	0.003
Glass hybrid	12 h	Dry environment-Cow’s milk	1.177	0.245	4.810	<0.001	<0.001
Glass hybrid	12 h	Dry environment-Goat milk	1.059	0.245	4.327	<0.001	<0.001
Glass hybrid	12 h	Artificial saliva-Cow’s milk	0.701	0.245	2.864	0.004	0.046
Glass hybrid	12 h	Tap water-Cow’s milk	1.075	0.245	4.391	<0.001	<0.001
Glass hybrid	12 h	Tap water-Goat milk	0.957	0.245	3.907	<0.001	0.001
Glass hybrid	12 h	Cow’s milk-Kefir	−0.689	0.245	−2.815	0.005	0.049
Glass hybrid	24 h	Dry environment-Artificial saliva	0.997	0.245	4.073	<0.001	<0.001
Glass hybrid	24 h	Dry environment-Cow’s milk	1.521	0.245	6.213	<0.001	<0.001
Glass hybrid	24 h	Dry environment-Goat milk	1.125	0.245	4.595	<0.001	<0.001
Glass hybrid	24 h	Dry environment-Kefir	0.866	0.245	3.537	<0.001	0.004
Glass hybrid	24 h	Tap water-Cow’s milk	1.102	0.245	4.502	<0.001	<0.001
Glass hybrid	24 h	Tap water-Goat milk	0.706	0.245	2.885	0.004	0.039
Composite	1 h	Artificial saliva-Cow’s milk	−0.717	0.245	−2.930	0.003	0.044
Composite	1 h	Tap water-Kefir	0.727	0.245	2.969	0.003	0.042
Composite	1 h	Cow’s milk-Kefir	0.869	0.245	3.549	<0.001	0.006
Composite	6 h	Artificial saliva-Cow’s milk	−0.793	0.245	−3.239	0.001	0.017
Composite	6 h	Tap water-Kefir	0.758	0.245	3.095	0.002	0.024
Composite	6 h	Cow’s milk-Goat milk	0.775	0.245	3.165	0.002	0.020
Composite	6 h	Cow’s milk-Kefir	1.021	0.245	4.171	<0.001	<0.001
Composite	12 h	Dry environment-Kefir	0.783	0.245	3.199	0.001	0.015
Composite	12 h	Artificial saliva-Cow’s milk	−0.810	0.245	−3.308	<0.001	0.012
Composite	12 h	Tap water-Kefir	0.860	0.245	3.512	<0.001	0.006
Composite	12 h	Cow’s milk-Goat milk	0.794	0.245	3.243	0.001	0.014
Composite	12 h	Cow’s milk-Kefir	1.131	0.245	4.621	<0.001	<0.001
Composite	24 h	Dry environment-Artificial saliva	0.903	0.245	3.689	<0.001	0.002
Composite	24 h	Dry environment-Goat milk	0.735	0.245	3.004	0.003	0.023
Composite	24 h	Dry environment-Kefir	1.173	0.245	4.792	<0.001	<0.001
Composite	24 h	Artificial saliva-Tap water	−0.746	0.245	−3.048	0.002	0.023
Composite	24 h	Artificial saliva-Cow’s milk	−0.908	0.245	−3.709	<0.001	0.002
Composite	24 h	Tap water-Kefir	1.016	0.245	4.150	<0.001	<0.001
Composite	24 h	Cow’s milk-Goat milk	0.740	0.245	3.023	0.003	0.023
Composite	24 h	Cow’s milk-Kefir	1.178	0.245	4.812	<0.001	<0.001

## Data Availability

The raw data supporting the conclusions of this article will be made available by the authors on request.

## Abbreviations

The following abbreviations are used in this manuscript:

CRIS	Checklist for Reporting In-Vitro Studies
L*	Lightness
a*	Red-Green Axis
b*	Yellow-Blue Axis
IADT	International Association of Dental Traumatology
ΔE	Color Difference
EMM	Estimated Marginal Mean
ML	Maximum Likelihood
SEM	Scanning Electron Microscopy
HBSS	Hank’s Balanced Salt Solution
